# Assessing the integrity of auditory processing and sensory memory in adults with cystinosis (*CTNS* gene mutations)

**DOI:** 10.1186/s13023-021-01818-0

**Published:** 2021-04-13

**Authors:** Ana A. Francisco, Alaina S. Berruti, Frederick J. Kaskel, John J. Foxe, Sophie Molholm

**Affiliations:** 1 Department of Pediatrics, Albert Einstein College of Medicine, Van Etten Building, Suite 1C, 1225 Morris Park Avenue, Bronx, NY 10461 USA; 2 Department of Neuroscience, Rose F. Kennedy Center, Albert Einstein College of Medicine, Bronx, NY USA; 3 Department of Neuroscience, The Ernest J. Del Monde Institute for Neuroscience, School of Medicine and Dentistry, University of Rochester, Rochester, NY USA

**Keywords:** EEG, Auditory evoked potential, Copy number variation, Event-related potential, N1, P2, Mismatch negativity, P3a, Lysosomal storage disorder

## Abstract

**Background:**

Cystinosis, a rare lysosomal storage disease, is characterized by cystine crystallization and accumulation within tissues and organs, including the kidneys and brain. Its impact on neural function appears mild relative to its effects on other organs, but therapeutic advances have led to substantially increased life expectancy, necessitating deeper understanding of its impact on neurocognitive function in adulthood. We previously demonstrated intact auditory sensory processing, accompanied by mild sensory memory difficulties, in children and adolescents with cystinosis.

**Methods:**

We investigated whether further progressive decrements in these processes would be observed in adults with cystinosis, comparing high-density auditory-evoked potential (AEP) recordings from adults with cystinosis (N = 15; ages: 19–38 years) to those of age-matched controls (N = 17). We employed a duration oddball paradigm with different stimulation rates, in which participants passively listened to regularly occurring standard tones interspersed with infrequently occurring deviant tones. Analyses focused on AEP components reflecting auditory sensory-perceptual processing (N1 and P2), sensory memory (mismatch negativity, MMN), and attentional orienting (P3a).

**Results:**

Overall, adults with cystinosis produced highly similar sensory-perceptual AEP responses to those observed in controls suggesting intact early auditory cortical processing. However, significantly increased P2 and P3a amplitudes and reduced MMN at slower stimulation rates were observed, suggesting mild-to-moderate changes in auditory sensory memory and attentional processing. While cognitive testing revealed lower scores on verbal IQ and perceptual reasoning in cystinosis, these did not correlate with the AEP measures.

**Conclusions:**

These neurophysiological data point to the emergence of subtle auditory processing deficits in early adulthood in cystinosis, warranting further investigation of memory and attentional processes in this population, and of their consequences for perceptual and cognitive function.

## Background

Cystinosis is a rare autosomal recessive condition caused by a bi-allelic mutation in the 17p13.2-located *CTNS* gene [1]. It is characterized by excessive intralysosomal storage and crystallization of cystine [2], which trigger significant system-wide damage in various tissues and organs [3]. Kidney dysfunction remains cystinosis’ primary clinical characteristic [4], but the development of renal replacement therapy and cysteamine, a cystine-depleting agent which stalls the progression of renal failure and protects extra-renal organs [5], has attenuated the significant renal complications associated with the condition. Consequently, the life expectancy for this population has dramatically increased [6]. It is now necessary to characterize the impact of cystinosis in adulthood, especially as it pertains to longer-term cognitive health.

Little is known about the long-term progression and sequelae of cystinosis and its treatments. Of particular interest is the characterization of brain function, given that central nervous complications (for instance, strokes, seizures, and consequent neurocognitive dysfunction) are among the additional concerns associated with disease evolution [7–9] and that abnormally high levels of cystine have been reported in multiple brain regions [10–12].

Studies investigating cognitive and neural function in adults with cystinosis are scarce and tend to be inconsistent. For example, two behavioral studies using the same verbal and visual learning/memory paradigm reported conflicting findings: While one suggested an overall decline in performance with age in cystinosis, indicating progressive dysfunction over the lifespan [13], a later study from the same group reported no differences between adults with and without cystinosis [14]. Frankel and Trauner argued that potential earlier treatment onset and overall advances in treatment might have prevented or reduced cognitive deficits in their sample. Electrophysiological (EEG) studies have likewise led to contradictory findings. One case study looked at auditory and somatosensory evoked potentials in a single adult female. Though no methodological details or specific results were included, typical neural activity was reported [15]. A conference paper reported an enhanced auditory-evoked potential (AEP) at around 200 ms after stimulus presentation for 14 individuals diagnosed with cystinosis (aged: 6 to 52 years) during a spatial localization task [16].

In a recent high-density electrophysiological study, our research group investigated auditory sensory-perceptual processing and sensory memory processes, specifically in children and adolescents with cystinosis. We used a simple auditory oddball paradigm whereby regular tones (1000 Hz, 100 ms duration, “standard” stimuli) were infrequently interrupted by a tone of the same frequency but of slightly longer duration (180 ms, “deviant” stimuli). This allows for assessment of basic sensory-perceptual processing by interrogating the AEP componentry evoked by the standard stimuli, and of auditory sensory memory, by interrogating the so-called mismatch negativity (MMN) component evoked by the duration deviant. In our young cystinosis cohort, no differences were found in basic auditory processing between the groups, but a deficit in sensory memory appeared to be present in those with cystinosis [17]. As such, differences in auditory processing in early-stage disease were subtle. A key question is whether these relatively subtle differences will progress to more severe deficits as individuals enter adulthood.

Here, we employed the same duration oddball paradigm in a sample of adults with cystinosis and age-matched controls and focused on AEP components indexing basic auditory cortical processing (N1 and P2; see e.g., [18–21]), sensory memory (MMN), and involuntary attentional orienting (P3a). As mentioned above, the MMN is elicited by a deviant stimulus presented within a stream of standard stimuli [22], and signifies violation of sensory memory based expectations [23–25]. When the P3a is similarly elicited by a deviant stimulus in an oddball paradigm, it is argued to represent the automatic orienting response to novel or salient stimuli and appears to originate from stimulus-driven, prefrontal attention mechanisms [26–29].

Considering earlier work suggesting associations between auditory sensory memory and working memory [30–32], we also tested working memory capacity and its association with the MMN. Additionally, given the consistently described discrepancies between verbal and non-verbal IQ in cystinosis [14, 33, 34], we measured verbal IQ and perceptual reasoning. Lastly, to account for the potential impact of number of renal transplants and cysteamine dosage on the amplitude of the components of interest, we tested for associations between these variables.

## Results

Table 1 shows a summary of the included participants’ demographics and performance on the WASI-II and on the Digit Span subtest of the WAIS-IV. Two-sample independent-means *t* tests were used to test for between-group differences. In cases in which the assumption of the homogeneity of variances was violated, *Welch* corrections were applied to adjust the degrees of freedom. Paired *t* tests were used to test for within-group differences. Statistical analyses revealed significant differences between the groups in IQ, with individuals diagnosed with cystinosis showing lower scores on verbal IQ and perceptual reasoning, but not on the Digit Span subtest (working memory). Both groups presented significantly lower perceptual reasoning scores when compared to verbal IQ.

**Table 1:** Characterization of controls and individuals with cystinosis included in the analyses: age, gender, IQ, and working memory

	Controls	Cystinosis	t test	Cohen’s d
Age	X = 26.94; SD = 6.49	X = 27.46; SD = 6.47	*t* = − 0.23, *df* = 29.48, *p* = .82	*d* = 0.08
Gender	6 males, 11 females	2 males, 13 females	–	–
Verbal IQ	X = 110.82; SD = 16.29	X = 96.40; SD = 8.77	*t* = 3.17, *df* = 25.14, *p* = .01	*d* = 1.10
Perceptual Reasoning	X = 104.18; SD = 13.77	X = 87.87; SD = 10.28	*t* = 3.82, *df* = 29.25, *p* = .01	*d* = 1.34
Working Memory	X = 9.59; SD = 2.29	X = 8.28; SD = 2.09	*t* = 1.65, *df* = 28.66, *p* = .11	*d* = 0.60
IQ: verbal versus p. reasoning				
NT			*t* = − 2.56, *df* = 16, *p* = .03	*d* = 0.89
Cystinosis			*t* = − 2.84, *df* = 14, *p* = .03	*d* = 0.44

**Fig. 1:**
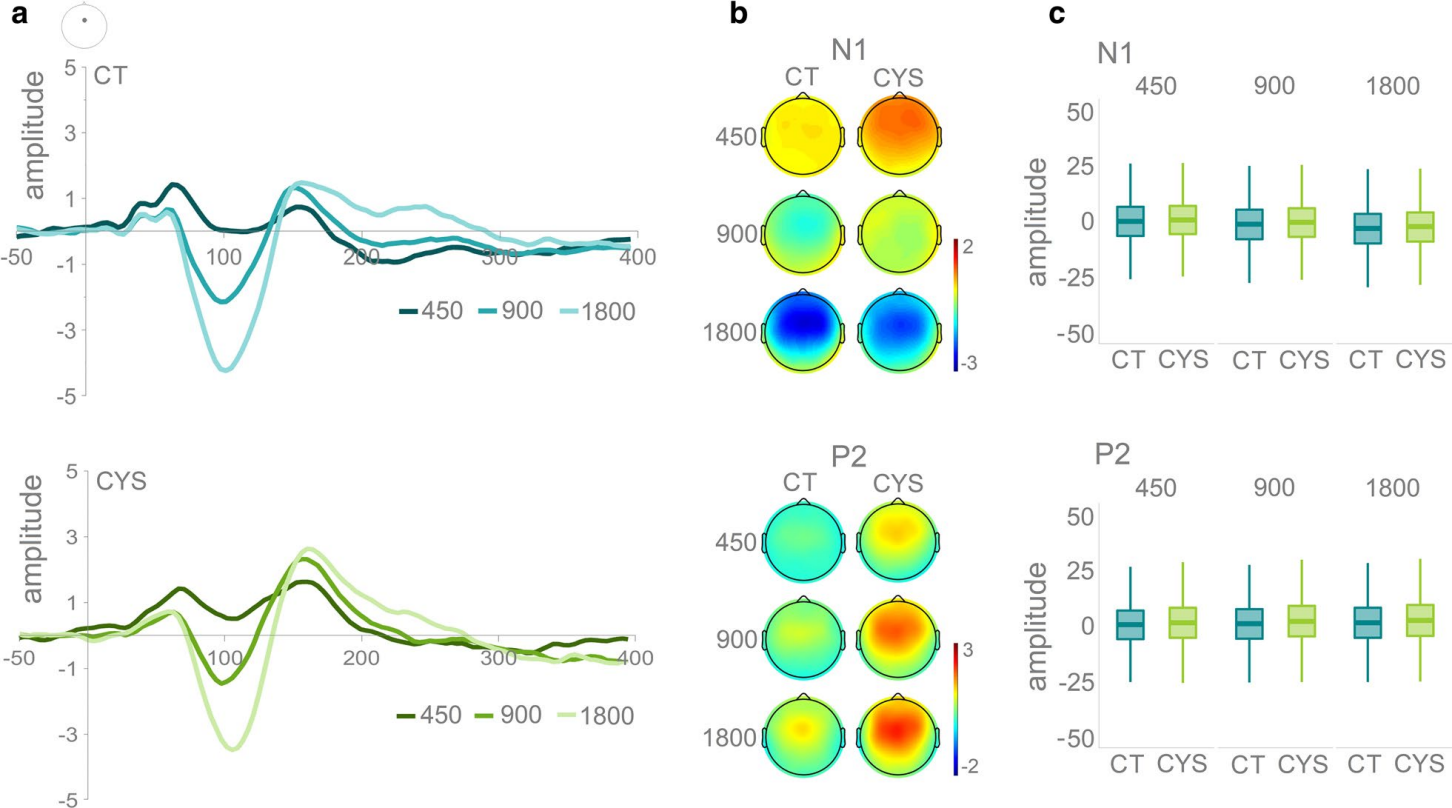
**a** Averaged AEPs per SOA for the standards per group at FCz. **b** Topographies for the N1 and the P2 time windows (standards only), organized from the shorter (450 ms) to the longer (1800 ms) SOA. **c** Boxplots showing distribution per group and SOA

Figure 1 shows the averaged AEPs and topographies for the N1 and P2 per SOA and by group. Mixed-effects models were implemented to analyze the EEG data, using the *lmer* function in the lme4 package [35] in R (Version 3.1.2, [36]). The models were run separately for the N1 and the P2 time windows. Mean amplitude at FCz was the numeric dependent variable. Only standard amplitudes were considered. Group (controls = − 0.5, cystinosis = 0.5) was a contrast-coded fixed factor, and SOA was a numeric fixed factor. Subject and trial were random factors. Models were fit using the maximum likelihood criterion. *P* values were estimated using Satterthwaite approximations [37].

In the N1 time window, there was an effect of SOA, with both 900 (*ß* = − 1.39, SE = 0.12, *p* = 0.01) and 1800 (*ß* = − 3.26, SE = 0.12, *p* = 0.01) evoking larger responses than 450 ms. No effects of group or of the interaction between group and SOA were observed. In the P2 time window, individuals with cystinosis presented larger responses than the controls (*ß* = 1.04, SE = 0.35, *p* = 0.01). There was also an effect of SOA, with both 900 (*ß* = 0.49, SE = 0.13, *p* = 0.01) and 1800 (*ß* = 0.98, SE = 0.13, *p* = 0.01) evoking larger responses than the 450 ms SOA.

**Fig. 2:**
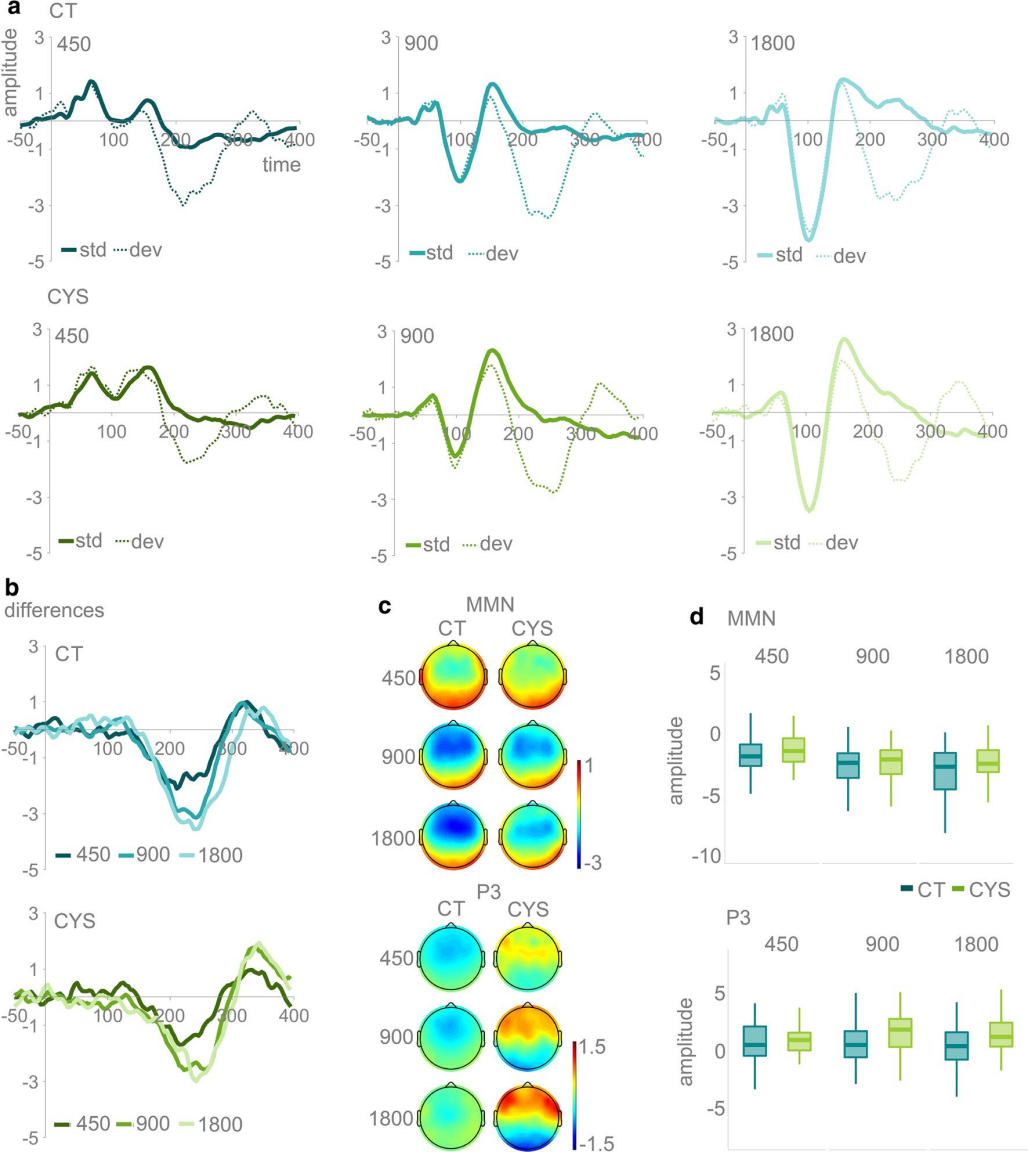
**a** Averaged AEPs per SOA and group for standards and deviants at FCz. **b** Differences (deviant-standards) per group and SOA at FCz. **b** Topographies for the MMN and the P3a time windows (differences), organized from the shorter (450 ms) to the longer (1800 ms) SOA; **c** Boxplots showing distribution per group and SOA for MMN and P3a (differences)

Figure 2 shows the averaged AEPs and topographies for the MMN and P3a per SOA and by group. Mixed-effects models were carried out as described for the N1 and P2 time windows. In the MMN time window, for the individuals with cystinosis, the difference between the 1800 and the 450 ms SOA was reduced (*ß* = 0.50, SE = 0.13, *p* = 0.01) when compared to controls. Post-hoc analyses revealed that this was mainly explained by a reduced MMN in the 1800 SOA in the cystinosis group (*ß* = − 0.91, SE = 0.43, *p* = 0.04). There was also an effect of SOA, with both 900 (*ß* = − 0.95, SE = 0.09, *p* = 0.01) and 1800 ms (*ß* = − 1.44, SE = 0.09, *p* = 0.01) SOAs evoking larger responses than the 450 ms SOA. In the P3a time window, when compared to their peers, individuals with cystinosis presented an increased amplitude for the 900 (*ß* = 0.57, SE = 0.16, *p* = 0.01) and 1800 ms (*ß* = 0.80, SE = 0.16, *p* = 0.01) SOAs. No effects of SOA were observed.

No significant correlations were found between cognitive function, number of renal transplants and cysteamine dosage, and neural responses across or within groups.

## Discussion

High-density EEG recordings during a passive oddball paradigm characterized basic auditory processing, sensory memory, and involuntary attention in a sample of adults with cystinosis. Although statistically significant differences were observed in the P2, MMN, and P3a amplitudes between the groups, these differences were quite subtle, and by-and-large adults with cystinosis presented highly similar neural responses compared to those of age-matched controls.

In the N1 time window, no detectable differences were found between groups, suggesting intact sensory transmission through the auditory system in individuals with cystinosis. Further indicative of intact basic auditory processing in cystinosis was the modulation of the N1 as a function of SOA (i.e. rate of presentation). This modulation, consistently described in the literature for the neurotypical population [38] as reflecting typical habituation [39, 40] and/or refractoriness [41, 42], was present in both control and cystinosis groups. These results were fully consistent with those we previously observed in children and adolescents with cystinosis [17], suggesting that basic auditory sensory-perceptual processing is maintained across development in this population and does not suffer from decline with age, in contrast to visual memory and learning [13]. Thirteen out of the fifteen individuals with cystinosis tested received a kidney transplant and thus these findings are not due to the inclusion of a non-representative, atypically healthy adult sample of cystinosis.

In the P2 time window, individuals with cystinosis presented increased amplitudes when compared to their peers, which was not observed in children and adolescents in our earlier study [17]. An enhanced P2 has been previously suggested in cystinosis in a proceedings paper [16], though in a sample with a significantly wider range of ages, and during a task focused on spatial selective attention. The authors argued that the larger amplitudes observed in cystinosis could be explained by anatomical factors such as a thinner skull, caused by renal osteodystrophy present in this population [16]. The amplitudes of the electrical potentials recorded on the scalp surface are affected by the conductive properties of the volume between the cortex and scalp surface: A greater electrical resistance of the volume (i.e., a thicker skull) results in reduced EEG amplitudes; a decreased electrical resistance of volume (i.e., a thinner skull) results in increased EEG amplitudes [43–45]. Skull thinness could thus be a potential explanation for the increased P2 in cystinosis. However, one would then expect a similar increase to be observed across all AEP sensory components, which was not the case in the current study. Such an increase should likewise be observed in children and adolescents, but no differences were found in P2 in our previous study testing children and adolescents.

Though often conceptualized in the context of the N1/P2 complex, P2 can be dissociated from the N1 experimentally, developmentally, and topographically (for a review, see [46]). The findings herein, suggesting no differences between the groups in the N1, but an increased P2 in cystinosis, are in accordance with a dissociation between these two components. The functional significance of the P2 is, however, poorly understood. In the context of oddball paradigms, P2 has been argued to reflect attention modulation [47], to index stimulus classification (whereby a stimulus is considered to be target or non-target) [48], and/or to be related to consolidation processes associated with auditory memory formation and learned relevance [49, 50]. Increased P2 amplitudes, however, have been mainly associated with attentional processes. While some argue that such enhancement may reflect a deficit in the capacity to withdraw attentional resources from stimuli [48] or the capture of more attention [51], others suggest that differences in P2 amplitude may simply be an indication of the involvement of different processes underlying attentional disengagement [46]. When compared to their age-matched peers, individuals with cystinosis might have engaged attention differently while passively listening to tones. The consequences of these potential differences warrant further investigation. An active oddball paradigm, for instance, would allow one to measure deviant detection rate and test whether those with increased P2 amplitudes are more or less accurate in detecting the deviant tones. Behaviorally, in standardized tasks and during behavioral observations, children and adolescents with cystinosis appear to present some level of impairment in the domain of attention [52–54], but no study has yet focused on this process during adulthood. Thorough behavioral and electrophysiological investigations of voluntary and involuntary attention in this population are thus warranted.

In the MMN time window, despite showing robust MMN responses across SOAs, individuals with cystinosis showed a slightly decreased response to the 1800 ms SOA when compared to their age-matched peers. In children and adolescents, such reductions were likewise observed, not only for the 1800 ms, but also for the 900 ms SOA, which we interpreted as a sign of reduced short-term auditory sensory memory [55, 56]. Hence, such difficulties might still be present in adulthood, though less markedly so. Auditory sensory memory, a preattentive memory system that allows an individual to retain traces of sensory information after the termination of the original stimulus [57], could impact subsequent processing in working memory [30], a conscious cognitive system responsible for the temporary holding, processing, and manipulation of information [58]. And, indeed, the MMN has been associated with performance in memory tasks in both neurotypical and clinical populations [30–32]. Here, however, and despite the differences found between the groups in the longer SOA MMN, individuals with cystinosis performed similarly to controls on a standardized behavioral working memory task. Thus, this warrants further investigation of the neurophysiology of sensory and working memory processes in this population, and consideration of their consequences for perceptual and cognitive function.

One of the express purposes of employing three rates of presentation in the current study was to parametrically tax the auditory sensory memory system. That is, when the tones are presented at a rapid rate (> 2 Hz; 450 ms SOA), this rapid presentation establishes a strong auditory sensory memory trace for the standard tones such that the longer duration deviants strongly “pop out”. As the rate slows (~ 0.5 Hz; 1800 ms SOA), the sensory memory representation of the ongoing standards tends to weaken, and the general thesis is that the longer deviants are less perceptible at these slower rates (i.e., they do not pop out as much). As can been seen from the MMN data herein, even at the slowest rate, a robust MMN was generated for both groups. In contrast, in data collected from individuals with Rett Syndrome using the same paradigm, an MMN was only detected at the fastest rate and was completely absent at the two slower rates [56]. Clearly then, this manipulation was not sufficient to assay potentially more modest differences in this clinically less affected group. The reader will also note that the duration deviant used here (180 ms vs. a 100 ms standard) is very large, and consequently highly perceptible. A potentially more sensitive test could involve the use of progressively less noticeable duration deviants, which would allow for more finely calibrated testing of these more subtle phenotypes in cystinosis (e.g. [59]). It is also worth noting that there are both hierarchically early sensory generators of the duration MMN, as well as higher-order cortical contributions from regions in the frontal and parietal lobes [60]. Given the current findings of intact early sensory processing, as represented by the wholly intact N1, it is possible that subtle deficits seen in the duration MMN at the slowest rate here may be specifically attributable to deficits in these higher-order cortical generators. It will fall to future work to determine if this is the case, but such a finding would point to the emergence of subtle higher-order deficits rather than early sensory processing.

Lastly, in the P3a time window, and similar to what was observed for the P2, individuals with cystinosis presented increased amplitudes for the 900 and 1800 ms SOA conditions. There are several interpretations of the role of the P3 component (see [61]). One common view is that it represents context updating when stimulus events require that the model of the environment be revised [62]. The value, significance, and relevance of the stimulus dictate the extent to which the updating process is activated [63]. Another view, and perhaps a better alternative in the context of passive paradigms, is that P3 is associated with event categorization: Each stimulus is evaluated and categorized as expected, unexpected, related to the task, etc., but with little controlled processing involved [64]. A larger P3 in cystinosis could then be suggestive of an easier categorization process [64]. One could argue that if attentional resources were allocated differently in cystinosis in earlier stages of information processing (which the increased P2 in this population could be indicative of) [65], the later categorization of the stimulus could have been facilitated in this group. An active oddball paradigm with behavioral outcomes will be useful in understanding the impact of the increased P3 on task performance. While we think it unlikely, an alternative explanation is that attention was differently engaged at the group level by the self-selected silent movie that participants watched during the experiment, with one group selecting a more engaging set of movies by chance than the other.

In summary, this study provides the first neural evidence of auditory sensory memory and involuntary attention differences in adults with cystinosis and pinpoints areas that warrant more research. It is nevertheless important to stress that despite the differences found between the groups, the adults with cystinosis tested here presented overall similar neural responses to controls. Some limitations should be noted. First, larger samples would have allowed for more detailed analyses focused on the associations between neural, cognitive, and behavioral outcomes and further investigation of the effects of cysteamine and its dosage on brain function. Given that compliance with cysteamine has been linked to better clinical outcomes [66], it could also be relevant to include a measure of compliance to treatment in future studies. Second, though all participants provided subjective reports of good health status at the time of testing, consideration of objective health history and current biomarkers will be useful in understanding the potential role of chronic and current illness in these group-level differences. Future research should include, for example, measures of renal function. Third, an active oddball paradigm would have been particularly useful in understanding the impact of the P2 and P3a amplitude differences in the successful detection of deviants. Lastly, our groups were not matched in terms of IQ, with the cystinosis group presenting significantly lower scores than the control group. Although such differences could have impacted the results, IQ is not typically associated with early sensory cortical responses, as others and we have shown[17, 67].

## Conclusions

We used high-density EEG recordings and a passive duration oddball paradigm to investigate basic auditory processing, sensory memory, and involuntary attention in a sample of adults with cystinosis. Our findings suggest intact early auditory processing, but mild-to-moderate changes in auditory sensory memory and attentional processing in cystinosis. Given that group differences appear to be stronger in our prior study in children, such changes do not seem to be due to chronicity and/or time taking medication. This work contributes to the understanding of how brain function is affected in cystinosis, and suggests future directions of research to advance such understanding. The knowledge that we gain through this work can be used to inform the development of appropriate intervention strategies designed to positively impact everyday functioning for individuals with cystinosis.

## Methods

### Participants

Fifteen adults with cystinosis (age range: 19–38 years old, all on cysteamine, drug that reduces the amount of cystine in the body) and 17 age-matched controls were recruited for this study. Hearing difficulties and current neurological problems were exclusionary criteria for the cystinosis group. Hearing problems, developmental and/or educational difficulties or delays, and neurological problems were exclusionary criteria for the control group. A hearing test (below 25 dB HL for 500, 1000, 2000, 4000 Hz) was performed on both ears using a Beltone Audiometer (Model 112). All individuals presented typical hearing thresholds. One individual with cystinosis was tested at an off-site location and, therefore, no hearing test was conducted. This individual reported normal hearing. All participants signed an informed consent approved by the Institutional Review Board of the Albert Einstein College of Medicine. Participants were monetarily compensated for their time. All aspects of the research conformed to the tenets of the Declaration of Helsinki.

### Experimental procedure and stimuli

Tests of cognitive function and EEG data were collected over a 2-day period. The Wechsler Abbreviated Scale of Intelligence, WASI-II [68] was used to assess verbal IQ and perceptual reasoning. The Digit Span subtest (Forward, Backward, and Sequencing measures) of the Wechsler Adult Intelligence Scale, WAIS-IV [69] was utilized to assess working memory. A traditional duration MMN oddball paradigm [17, 56, 70] was employed. Participants watched a muted movie of their choice on a laptop (Dell Latitude E6430 ATG or E5420M) while sitting in a sound- and electrically-shielded booth (Industrial Acoustics Company Inc, Bronx, NY). Using a pair of Etymotic insert earphones (Etymotic Research, Inc., Elk Grove Village, IL, USA), participants listened to regularly (85%) occurring standard tones intermixed with infrequently occurring deviant ones (15%). These were 75 dB SPL tones with a frequency of 1000 Hz and a rise and fall time of 10 ms. Whereas standard tones had a duration of 100 ms, deviant tones were 180 ms in duration. To yield an MMN, a random oddball configuration was employed, except that at least two standards preceded a deviant. Three blocked stimulus onset asynchrony (SOA) conditions were used: 450 ms, 900 ms and 1800 ms. Each SOA was presented in 4 min long blocks (counterbalanced across participants), composed of 500, 250 or 125 trials respectively. Participants were presented with 14 blocks (2 * 450 ms, 4 * 900 ms and 8 * 1800 ms), resulting in a possible 1000 trials (and 150 deviants) per SOA.

### Data acquisition and analysis

EEG data were acquired continuously at a sampling rate of 512 Hz from 71 locations using 64 scalp electrodes mounted on an elastic cap and seven external channels (mastoids, temples, and nasion) (Active 2 system; Biosemi^tm^, The Netherlands; 10-20 montage). Preprocessing was done using the EEGLAB toolbox (version 2019; Delorme & Makeig, 2004) for MATLAB (version 2019; MathWorks, Natick, MA) (full pipeline can be accessed at: github.com/DouweHorsthuis/EEG_pipeline_auditory_oddball_duration_tones_passive).

Data were downsampled to 256 Hz, re-referenced to TP8 and filtered using a 1 Hz high pass filter (0.5 Hz transition bandwidth, filter order 1690) and a 45 Hz low pass filter (5 Hz transition bandwidth, filter order 152). Both were zero-phase Hamming windowed sinc FIR filters. Bad channels were automatically detected based on kurtosis measures and rejected after visual confirmation (number of channels excluded: controls: M = 5, SD = 1; cystinosis: M = 5, SD = 3). Artifacts resulting from eye blinks and saccades were removed by running an Independent Component Analysis (ICA). After ICA, the previously excluded channels were interpolated, using the spherical spline method. Data were segmented into epochs of − 100 to 400 ms using a baseline of − 50 to 0 ms. These epochs went through an artifact detection algorithm (moving window peak-to-peak threshold at 120 µV). All subjects had trial rejection rates below 30%. No significant differences were found between the number of trials included in the analyses per group (standards: controls: M = 2527, SD = 77; cystinosis: M = 2412, SD = 262, *t* = 1.62, *df* = 16.14, *p* = 0.12, *d* = 0.59; deviants: controls: M = 444, SD = 17; cystinosis: M = 425, SD = 46, *t* = 1.47, *df* = 17.59, *p* = 0.16, *d* = 0.53).

The definition of the time windows of interest was based on the typical time of occurrence of the components, and on visual confirmation that amplitudes were maximal in these intervals. Mean amplitude for the N1 was measured between 90 and 130 ms, P2 was measured between 150 and 180 ms, MMN between 200 and 260 (100 to 160 ms post deviance onset), and P3a between 310 and 350 ms. Mean amplitude measures (trial-by-trial for N1 and P2 for standards only, difference in amplitude between standards and deviants for MMN and P3a) were taken at FCz, where signal was at its maximum for both groups. These amplitudes were used for between-groups statistics and correlations. Correlation analyses were computed across and between groups per component. Pearson correlations were performed for most of the variables, given that their distributions were not significantly different from the normal distribution, as tested by the *Shapiro-Wilks* Normality test [71], implemented using the *shapiro.test* function of the *stats* package in R [36]: verbal IQ: *W* = 0.94, *p* = 0.06; perceptual reasoning: *W* = 0.97, *p* = 0.56; working memory: *W* = 0.95, *p* = 0.15; N1 amplitude: *W* = 0.95, *p* = 0.19; P2 amplitude: *W* = 0.97, *p* = 0.57; P3a amplitude: *W* = 0.96, *p* = 0.37. The distributions of cysteamine dosage (*W* = 0.76, *p* = 0.01), number of transplants (*W* = 0.68, *p* = 0.01), and MMN amplitude (*W* = 0.92, *p* = 0.02) were different from normal distribution and thus Spearman correlations were instead used for these variables. All *p-values* (from post-hoc tests and correlations) were submitted to *Holm-Bonferroni* corrections for multiple comparisons [72], using the *p.adjust* of the *stats* package in R [36].

## Data Availability

The datasets used and/or analyzed during the current study are available from the corresponding author on reasonable request.

## Abbreviations

EEG: Electrophysiological
AEP: Auditory-evoked potential
MMN: Mismatch negativity
CT: Controls
CYS: Cystinosis
